# Quiz-Compends—Gynecology

**Published:** 1904-02

**Authors:** 


QUIZ-COMPENDS—GYNECOLOGY. By William H. Wells, M. D. Third revised edition, enlarged, with 145 illustrations. Published by P. Blakiston's Son & Co., 1012 Walnut Street, Philadelphia.

This the third edition has been completely revised up to date, and contains much new in the way of treatment especially. The sales of the former editions of this practical little work insures the reception of the present one. J. M. L.

